# A(H9N2) influenza viruses associated with chicken mortality in outbreaks in Algeria 2017

**DOI:** 10.1111/irv.12675

**Published:** 2019-09-03

**Authors:** Trushar Jeevan, Daniel Darnell, El Alia Gradi, Yasmine Benali, Redhouane Kara, Djamel Guetarni, Adam Rubrum, Patrick J Seiler, Jeri Carol Crumpton, Richard John Webby, Fawzi Derrar

**Affiliations:** ^1^ Department of Infectious Diseases St Jude Children's Research Hospital Memphis TN USA; ^2^ National Influenza Centre Viral Respiratory Laboratory Algiers Algeria; ^3^ Laboratory of Veterinary Pathology and Cytology Institut Pasteur of Algeria Algiers Algeria; ^4^ Veterinary Practitioner Blida Algeria; ^5^ Veterinary Department University Saad Dahlab Blida Algeria

**Keywords:** Algeria, H9N2, influenza

## Abstract

In late 2017, increased mortality was detected in chicken farms in Algeria undergoing A(H9N2) influenza outbreaks. Analysis of viruses isolated from affected farms showed that they were monophyletic, were of the G1 hemagglutinin (HA) lineage, and were antigenically and genetically similar to viruses detected contemporaneously in other countries in Northern Africa and the Middle East. The virus was able to spread via contact transmission between ferrets but did not cause disease in intravenously inoculated chickens.

A(H9N2) viruses are arguably the most common subtype of influenza virus detected in poultry populations globally. The virus is endemic in poultry in multiple regions of the world including Asia and the Middle East.^1^ Although considerable genotypic heterogeneity is present, the HA genes from the A(H9N2) viruses most frequently isolated from gallinaceous poultry fall into three dominant genetic groups represented by A/quail/Hong Kong/G1/97 (G1 lineage), A/chicken/Hong Kong/Y280/97 (Y280 lineage), and A/chicken/Beijing/1/94 (Ck/Bei lineage).^2^ While the geographic range of the Y280 and Ck/Bei lineages has remained relatively confined, viruses of the G1 lineage spread from Southeast Asia to the Indian subcontinent in the late 1990s,^3^ through the Middle East soon after,^4^ and more recently into Northern Africa.^5^ In Africa, widespread reports of A(H9N2) viruses in Egypt began in 2011,^6^ although other studies suggested that the virus was likely present earlier^4^, ^7^ and quickly became endemic cocirculating with A(H5N1) viruses.^8^ It appeared that a similar time frame likely existed for A(H9N2) viruses in Libya^4^ and Tunisia,^9^ although lack of longitudinal regular surveillance, as was implemented in Egypt, makes it difficult to determine the full impact of these viruses in these countries. Further, A(H9N2) viruses were detected in chickens in Morocco in 2016.^5^

As well as impacting veterinary health, A(H9N2) viruses have also been sporadically isolated from humans^10^ and represent a continuous zoonotic and potentially pandemic threat.^11^, ^12^ Studies have also shown that a small number of amino acid changes can impart sustained ferret airborne transmission to an A(H9N2) virus, a phenotype considered required of a pandemic virus.^13^ Due to these concerns, continued monitoring of the spread and nature of A(H9N2) viruses represents valuable activities for veterinary and human health. It was, thus, with much interest that A(H9N2) viruses were first detected in Algeria in late 2017 associated with poultry die‐off. Several poultry flocks from the region of Fouka experienced an unusual increase in mortality (peaking at 57%) with birds of 23‐26 days of age. Three of the affected farms were monitored during the period from October to November 2017; two were vaccinated as routinely performed against Newcastle, infectious bronchitis, and Gumboro disease. The third farm was vaccinated against the previous pathogens as well as with a monovalent H9N2 vaccine; this farm had a reduced mortality of 17%‐34%. Necropsies showed that birds were congested, which was particularly severe in the visceral organs such as trachea, lungs, kidneys, liver, and pancreas. Congestion and petechial hemorrhages were observed in the serous membrane of the intestine. Congestive and hemorrhagic conjunctivitis was observed, and fibrinous oculonasal discharge with infraorbital sinusitis was noticed. The tracheal mucosa was severely congested and edematous. Formation of fibrinous casts in the laryngo‐tracheal junction was also observed.

HA and neuraminidase (NA) gene sequences were generated from A(H9N2) viruses isolated from broiler‐chicken tracheal swabs by inoculation into 10‐day‐old embryonated hens’ eggs followed by incubation at 35°C for 48 hours. These sequence data (Genbank accession numbers MK240222‐MK240305) showed that all of the viruses belonged to the G1 lineage of A(H9N2) virus and were closely related to viruses previously detected in Morocco, Burkina Faso, and United Arab Emirates (Figure 1). The high similarity of the HA and NA sequences from individual Algerian viruses (99%‐100% identity) suggested a single‐source outbreak followed by local spread. Full genome sequencing was completed for five of the viruses, again confirming high similarities amongst outbreak viruses and viruses previously described from Northern Africa and the Middle East (data not shown).

**Figure 1:**
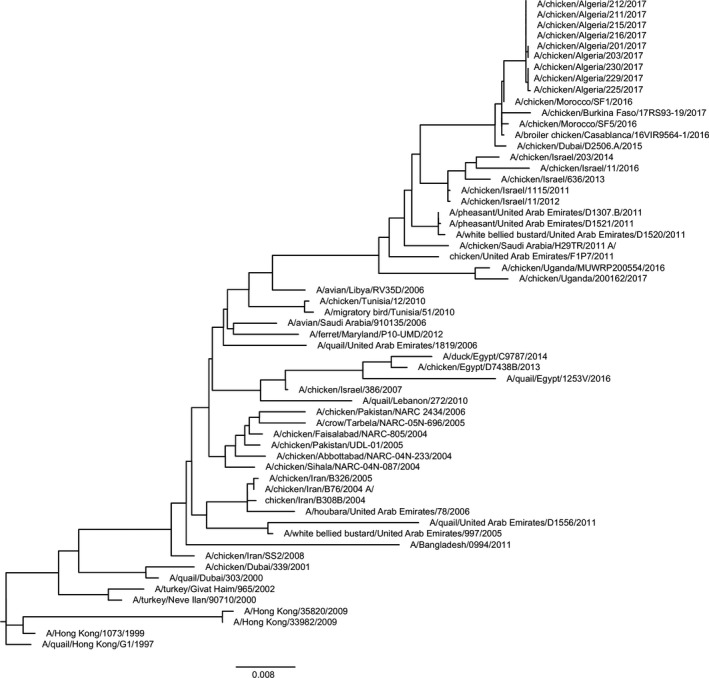
Phylogenetic analysis of H9 hemagglutinin (HA) genes. The hemagglutinin gene segments from all studied viruses and a selection of viruses present in GenBank were aligned in MUSCLE. Neighbor‐joining trees were constructed using the full nucleotide coding sequences of each HA gene segment using RAxML. RAxML was called using the rate heterogeneity model GTRGAMMA for 1000 reps. Evolutionary distances were computed using the Poisson correction method

To determine the antigenic properties of the isolated viruses in comparison with A(H9N2) reference viruses and to viruses from Northern Africa and the Middle East, hemagglutination inhibition (HAI) assays with 0.5% chicken red blood cells were conducted. The results of these assays showed that the 15 Algerian viruses tested, as expected from high sequence similarities, were antigenically similar to each other (Table 1). While the Algerian viruses reacted poorly to post‐infection antiserum produced to the prototypical G1 lineage virus A/Hong Kong/1073/97, they reacted well to a number of more recent G1 viruses including those isolated in Bangladesh and Uganda (Table 1).

**Table 1:** Hemagglutination inhibition assay of A(H9N2) viruses from Algeria and the surrounding region

	Clade	Bd/0994	CK/Egypt/D7438B	CK/Uganda/200162	CK/Dubai/339
Reference antigens					
A/Bangladesh/0994/2011	G1	**1280**	640	640	160
A/chicken/Egypt/D7438B/2013	G1	1280	**1280**	2560	640
A/chicken/Uganda/200162/2017	G1	1280	1280	**1280**	160
A/chicken/Dubai/339/2001	G1	320	320	320	**640**
Test antigens					
A/chicken/Algeria/216/2017	G1	320	640	320	320
A/chicken/Algeria/203/2017	G1	1280	1280	1280	320
A/chicken/Algeria/225/2017	G1	80	160	160	80
A/chicken/Algeria/230/2017	G1	640	640	640	160
A/chicken/Algeria/204/2017	G1	640	ND*	640	ND
A/chicken/Algeria/205/2017	G1	640	ND	640	ND
A/chicken/Algeria/209/2017	G1	640	ND	640	ND
A/chicken/Algeria/210/2017	G1	640	ND	640	ND
A/chicken/Algeria/214/2017	G1	640	ND	640	ND
A/chicken/Algeria/219/2017	G1	640	ND	640	ND
A/chicken/Algeria/221/2017	G1	320	ND	320	ND
A/chicken/Algeria/222/2017	G1	640	ND	640	ND
A/chicken/Algeria/224/2017	G1	640	ND	1280	ND
A/chicken/Algeria/228/2017	G1	640	ND	640	ND
A/chicken/Algeria/229/2017	G1	640	ND	1280	ND
A/chicken/Egypt/F12170A/2016	G1	160	320	320	40
A/quail/Dubai/303/2000	G1	160	40	40	160
A/pheasant/UAE/D1307.B/2011	G1	160	80	320	10

* ND = not determined.

*Note*: Titers in bold represent homologous titers.

A(H9N2) viruses have been postulated to have an elevated zoonotic risk due to the ability of some viruses to bind to sialic acid receptors in a terminal alpha 2‐6 confirmation,^14^ the confirmation preferred by mammalian influenza viruses including those adapted to humans.^15^ A number of human A(H9N2) infections have indeed been identified.^10^ In order to better assess the zoonotic threat posed by the Algerian A(H9N2) viruses, a ferret transmission experiment was conducted. Three adult male ferrets (*Mustela putorius furo*) (donor animals), 4 to 6‐month‐old, serologically negative for influenza and purchased from Triple F Farms, Bradford County, Pennsylvania, were directly inoculated with 1 mL of 1 x 10^6^ EID_50_ of A/chicken/Algeria/216/2017. Twenty‐four hours later, a direct and an airborne contact animal were introduced to each donor. Virus was titrated from nasal wash collections on days 3, 5, and 7 post‐inoculation from all three donor ferrets (Figure 2A). Peak titers ranged from 4.8 to 5.6 log10 TCID_50_/mL and were observed on day 3 post‐infection. One of the three ferrets had cleared the virus by day 7, the last day of nasal wash collections, although titers were also reduced from peak in both other animals. Three additional animals were similarly infected, and virus replication in the respiratory tract determined on day 5 post‐infection. Virus was recovered from nasal turbinate, trachea, and bronchi of all 3 ferrets with only trace amount of virus detected in lung samples; the highest titers were observed in turbinate and trachea tissue (Figure 2B). Correspondingly, no gross pathology was detected in the lungs of these animals. In direct contact animals, virus was recovered from nasal washes from two of the three ferrets on days 5 and 7 post‐inoculation, while no virus was recovered from the other ferret on any of the days sampled. None of the airborne contacts shed virus on any of the days samples were collected. All virologic data were further confirmed by serology. Infected animals had homologous HAI titers of 640, 640, and 1280, direct contacts had titers of 640, 640, and <10, and airborne contacts had titers of <10, <10, and <10. These data show that the Algerian A(H9N2) viruses are able to infect ferrets with replication limited primarily to the upper respiratory tract and transmit to direct contact ferrets but not to airborne contact animals. Minimal weight loss (<3% of starting body weight) was observed in donor ferrets, but no other clinical signs (neurologic symptoms, loss of appetite, sneezing, temperature changes, or lethargy) of infection were detected in any of the donor or contact ferrets.

**Figure 2:**
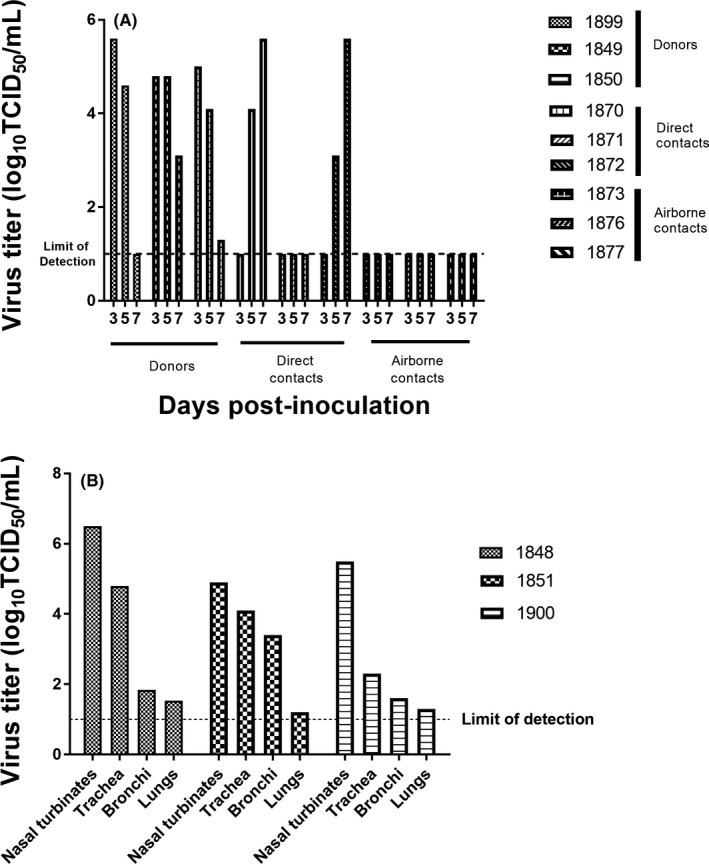
Viral loads in nasal washes (A) and tissues (B) from A(H9N2) virus infected ferrets. Nasal wash titers from each animal are shown for days 3, 5, and 7 post‐infection. Donor, direct contact, and airborne contact groups are indicated. Tissues in panel B were collected at day 5 post‐infection from a separate group of infected animals

Influenza viruses cause sporadic and endemic outbreaks in avian species across the globe. While severe disease in the avian host is most often associated with the highly pathogenic H5 and H7 viruses with additional amino acids in the HA cleavage site, mortality can also be associated with viruses of other subtypes. Although we did not have access to complete field epidemiologic data, these outbreaks were associated with deaths in affected flocks. Due to this associated mortality, and despite the presence of a typical low pathogenic avian influenza virus motif at the H9 connecting peptide, we conducted an intravenous pathogenicity index (IVPI) assay. No disease signs were observed, and the IVPI score for this virus was 0. The lack of disease symptoms in intravenously inoculated chickens suggests that field mortality was probably due to a combination of factors likely including other pathogens.

We could isolate a number of genetically and antigenically similar G1 lineage A(H9N2) viruses from birds in the affected farms. The similarity of the viruses isolated from different farms suggests a common source of infection and spread of the virus locally as opposed to outbreaks caused by multiple different endemic or imported viruses. A(H9N2) viruses have not been previously reported in Algeria, although G1 viruses have been detected in Northern Africa and the Middle East for at least a decade. The 2017 Algerian viruses had HA genes that were genetically most similar to viruses detected in Burkina Faso, Morocco, and United Arab Emirates from 2015 to 2017 suggesting dissemination of these viruses across Northern and Western Africa perhaps from a Middle Eastern source. This sublineage of virus is distinct from that which contains the endemic A(H9N2) viruses in Egypt, and it is unlikely that the Algerian viruses spread from that country. A(H9N2) viruses detected in Uganda in 2017 shared a more recent common ancestor with the Algerian viruses, but it was clear that one was not directly the source of the other.

A(H9N2) viruses are endemic in poultry populations in many regions of the world. Although there are insufficient longitudinal data to provide conclusive evidence, the identification of genetically similar A(H9N2) viruses in multiple countries of Africa suggests their further spread and entrenchment in the continent. Although these viruses have no characteristics suggestive of elevated pathogenic or zoonotic threat over other G1 lineage viruses, their endemicity would be an unwanted outcome with impact for veterinary and potentially human health.
